# Phospholipid-Gold Nanorods Induce Energy Crisis in MCF-7 Cells: Cytotoxicity Evaluation Using LC-MS-Based Metabolomics Approach

**DOI:** 10.3390/biom11030364

**Published:** 2021-02-27

**Authors:** Lina A. Dahabiyeh, Nouf N. Mahmoud, Mohammad A. Al-Natour, Laudina Safo, Dong-Hyun Kim, Enam A. Khalil, Rana Abu-Dahab

**Affiliations:** 1 Department of Pharmaceutical Sciences, School of Pharmacy, The University of Jordan, Amman 11942, Jordan; 2 Department of Pharmacy, Faculty of Pharmacy, Al-Zaytoonah University of Jordan, Amman 11733, Jordan; 3 Department of Pharmaceutics and Pharmaceutical Technology, The Faculty of Pharmacy and Medical Sciences, University of Petra, Amman 11196, Jordan; mohammad.alnatour@uop.edu.jo; 4 Centre for Analytical Bioscience, Advanced Materials and Healthcare Technologies Division, School of Pharmacy, University of Nottingham, Nottingham NG7 2RD, UK; Laudina.Safo1@nottingham.ac.uk (L.S.); dong-hyun.kim@nottingham.ac.uk (D.-H.K.); 5 Department of Pharmaceutics and Pharmaceutical Technology, School of Pharmacy, The University of Jordan, Amman 11942, Jordan; ekayoub@ju.edu.jo; 6 Department of Biopharmaceutics and Clinical Pharmacy, School of Pharmacy, The University of Jordan, Amman 11942, Jordan; abudahab@ju.edu.jo

**Keywords:** gold nanorods, metabolomics, LC-MS, MCF-7, amino acids, energy metabolism, TCA cycle

## Abstract

Phospholipid-modified gold nanorods (phospholipid-GNRs) have demonstrated drastic cytotoxicity towards MCF-7 breast cancer cells compared to polyethylene glycol-coated GNRs (PEG-GNRs). In this study, the mechanism of cytotoxicity of phospholipid-GNRs towards MCF-7 cells was investigated using mass spectrometry-based global metabolic profiling and compared to PEGylated counterparts. The results showed that when compared to PEG-GNRs, phospholipid-GNRs induced significant and more pronounced impact on the metabolic profile of MCF-7 cells. Phospholipid-GNRs significantly decreased the levels of metabolic intermediates and end-products associated with cellular energy metabolisms resulting in dysfunction in TCA cycle, a reduction in glycolytic activity, and imbalance of the redox state. Additionally, phospholipid-GNRs disrupted several metabolism pathways essential for the normal growth and proliferation of cancer cells including impairment in purine, pyrimidine, and glutathione metabolisms accompanied by lower amino acid pools. On the other hand, the effects of PEG-GNRs were limited to alteration of glycolysis and pyrimidine metabolism. The current work shed light on the importance of metabolomics as a valuable analytical approach to explore the molecular effects of GNRs with different surface chemistry on cancer cell and highlights metabolic targets that might serve as promising treatment strategy in cancer.

## 1. Introduction 

Nanoparticles (NPs) are new promising agents with wide biomedical applications including diagnostic, therapeutic, and drug delivery systems. Gold nanoparticles (GNPs), and particularly non-spherical GNPs such as gold nanorods (GNRs), exhibit unique properties which make them attractive therapeutic agents in nanomedicine. Anticancer properties of GNRs have been extensively investigated either as potential carriers of chemotherapeutic agents, or due to their intrinsic photothermal properties [1,2]. However, the current literature is mainly focusing on highlighting the physicochemical properties and the effect of GNPs on the living systems with little information on the consequences of their exposure on the cellular pathways. 

Currently, omics techniques, including genomics, transcriptomics, proteomics, and metabolomics, are widely applied in biomarker discovery, toxicology, and medical diagnostics [3,4]. Among omics approaches, metabolomics aims to identify and quantify the complete set of small biomolecules (e.g., amino acids, lipids, carbohydrates, alkaloids, and nucleic acids) within a biological system [5,6]. Therefore, metabolomics is considered a crucial analytical tool to obtain new valuable insights into the biology of organisms and to highlight changes in response to a specific stimuli [7]. Global metabolomics combines the use of state-of-the-art analytical techniques with advanced statistical analysis tools to comprehensively detect the changes in the metabolites profile of cells. Two main analytical platforms are used for this purpose: nuclear magnetic resonance (NMR) and mass spectrometry (MS) [8,9,10]. MS-based metabolomics in particular have witnessed tremendous technical and data processing advancements in recent years. Additionally, it offers high sensitivity, improved dynamic range, and high mass resolution that enable accurate and reliable analysis of diverse classes of metabolites even for complex biological samples such as tissues and cells [11,12,13].

Currently, metabolomics provides a promising analytical tool well suited to allowing in-depth assessment of the effect of various drugs and therapeutics on different types of biological samples and uncovering the underlying molecular mechanisms associated with their exposures. The metabolomics approach has been employed to evaluate toxicity induced by various NPs in human lung epithelial cells [14], A459 and H1299 lung carcinoma cell lines [15], human-like THP-1 macrophages [16], and yeast [17]. Moreover, metabolomics has been used to study the nanotoxicological effect of GNPs and functionalized GNPs in Wistar rats [18], Caco-2 cells [6], human hepatocellular carcinoma cell line C3A [19], and HeLa cells [20].

Recently, we have reported the high cytotoxicity of phospholipid-modified gold nanorods (phospholipid-GNRs) against MCF-7 breast cancer cells enhancing significant apoptotic events in addition to necrosis [21]. Interestingly, phospholipid-GNRs demonstrated significantly higher cytotoxicity to MCF-7 cells compared to polyethylene glycol (PEG)-coated GNRs (PEG-GNRs) over all tested concentrations [21]. Therefore, in the current study, we applied a cell-based global metabolic profiling using liquid chromatography-mass spectrometry (LC-MS) to provide further insights into the mechanism of cytotoxicity of phospholipid-GNRs on MCF-7 breast cancer cell line compared to PEG-GNRs.

## 2. Materials and Methods

### 2.1. Synthesis and Characterization of GNRs

GNRs were synthesized and modified with thiolated polyethylene glycol (PEG-SH) and 1,2-distearoyl-sn-glycero-3-phosphoethanolamine-N-PEG-SH to obtain PEG-GNRs and DSPE-PEG-GNRs (phospholipid-GNRs), respectively, and fully characterized as described previously [21]. Briefly, the synthesis of the nanorods was performed by producing a seed solution by mixing cetyltrimethylammonium bromide solution, 0.20 M (CTAB, Sigma Aldrich, Saint Louis, MO, USA) with chloroauric acid (0.005 M, HAuCl_4_, Sigma Aldrich, Saint Louis, MO, USA). Then 0.60 mL of ice-cold sodium borohydride solution (0.010 M, NaBH_4_, Sigma Aldrich, Saint Louis, MO, USA) was added to the mixture until a honey-colored solution was observed. The growth solution was prepared by dissolving sodium oleate and CTAB in 250 mL of hot water (~50°C), which was allowed to cool to 30 °C before adding 18 mL of silver nitrate (4 mM, AgNO_3_, Sigma Aldrich, USA). After that, 250 mL of HAuCl_4_ (1 mM) was added to the mixture and stirred for 90 min until it turned into colorless solution. Next, ascorbic acid (64 mM) and 0.8 mL of the seed solution were added to the growth solution. The resultant mixture was left for 48 h at 30 °C and the colorless solution turned into dark orange-brown solution. Double-round centrifugation of GNRs suspensions was performed for purification and the pellets were dispersed in milli-Q water. GNRs and the functionalized-GNRs were characterized by optical absorbance, particles size, and zeta potential. The surface conjugation of phospholipid into GNRs was confirmed by proton nuclear magnetic resonance (^1^H-NMR), Fourier transformed-infrared (FT-IR) spectroscopy, and thermogravimetric analysis (TGA) as described previously [21,22].

### 2.2. Treatment of MCF-7 Cells with PEG-GNRs and DSPE-PEG-GNRs and Metabolite Extraction

MCF-7 cells were seeded in 100 mm × 20 mm cell culture Petri dishes (Corning^®^, USA) in ten replicates for each condition at a density of 25 × 10^3^ cell per cm^2^ and incubated in 7 mL fully supplemented medium for 48 h. After reaching 80–90% confluence, cells were treated with 0.05 nM and 0.1 nM of phospholipid-GNRs and PEG-GNRs, respectively, while control cells were left without any treatment. After four hours of incubation, the treatment was removed, and the cells were incubated with complete RPMI media for 24 h. At the end of the incubation, the media were removed and the cells were briefly washed once with pre-warmed PBS (37 °C). Cellular metabolite extraction was performed as follows. Cells were quenched with 0.5 mL pre-cooled absolute methanol followed by cells scraping (over dry ice) and transferring the cell suspension into fresh 2 mL Eppendorf tubes. A volume of 500 µL chloroform (HPLC grade, Fisher Scientific, Loughborough, UK) was added, and the cell suspension was vortexed vigorously for 1 h at 4 °C, followed by the addition of 300 µL water (HPLC grade, Fisher Scientific, UK). The metabolites extract was centrifuged at 16,000× *g* for 10 min at 4 °C and the aqueous supernatant layer was removed and dried at room temperature using vacuum centrifuge evaporator. The dried aqueous cellular metabolite extract was stored at −80 °C until metabolite profiling using LC-MS. In the current work, only aqueous metabolites were analyzed using LC-MS. Non-aqueous metabolites (organic layer) was stored at −80 °C for future lipidomics study.

### 2.3. Metabolite Profiling Using Liquid Chromatography-Mass Spectrometry (LC-MS)

Before LC-MS analysis, aqueous metabolite extracts were reconstituted in 70 µL of pre-cooled methanol. Metabolites profiling was carried out using a Thermo Fisher Scientific Accela HPLC system coupled to an Orbitrap Exactive mass spectrometer (Thermo Fisher Scientific, Hemel Hempstead, UK). Metabolites were first separated using ZIC-pHILIC (150 × 4.6 mm id, 5 µm column from Merck Sequant, Watford, UK) and a mobile phase of 20 mM ammonium carbonate as solvent A, and 100% acetonitrile (LC-MS grade, Fisher Scientific, UK) as solvent B. Compounds were separated by linear gradient (20% A (0 min) to 95% A (15 min)) with a flow rate of 300 µL/min as described previously [15]. Separated metabolites were analyzed in full MS scan in the range of *m*/*z* 70 to 1400 with 50,000 resolution under both positive and negative ionization modes. Capillary and probe temperatures were maintained at 275 °C and 150 °C, respectively. A pooled quality control (QC) sample was prepared by mixing 10 µL of each sample in order to assess the performance of the LC-MS instrument. QC samples were injected every 10 samples, and the coefficient of variability (CV) was calculated for all mass ions to ascertain system suitability and stability [23]. A mixture of 250 authentic standards covering the most common and relevant metabolite groups was run before sample analysis to aid in metabolite identification. 

### 2.4. Data Analysis and Metabolite Identification 

Raw LC-MS data from the three study groups (control untreated MCF-7 cells and phospholipid-GNRs-, and PEG-GNRs-treated MCF-7 cells) were acquired using Xcalibur v2.1 software (Thermo Scientific, Hemel Hemstead, UK). Raw data were processed for untargeted peak picking with XCMS [24], while peak matching and related peak annotation were performed using mzMatch to assign each mass ion with the corresponding exact mass and retention time [25]. IDEOM was used for noise filtering and putative metabolite identification using default parameters [26]. 

Metabolites were identified, using IDEOM, either with level 1 or level 2 metabolites identification in accordance with the metabolomics standards initiative [27,28]. Metabolites with accurate masses and retention times that matched the corresponding analyzed authentic standards were identified with level 1, while in the case of absence of standards, metabolites were putatively identified (level 2) based on the accurate mass and the predicted retention times.

For univariate analysis, the processed raw data were uploaded to MetaboAnalyst version 4.0 [29,30] and one-way ANOVA was carried out to analyze the significance in the profile of the mass ions among the three groups. False discovery rate (FDR)-corrected *p*-value of less than 0.05 was considered significant.

Multivariate analysis was carried out using SIMCA-P v14.1 (Umetrics, Umea, Sweden) [31]. For modeling the differences between the studied groups, the datasets were normalized to their total sample median, log-transformed, and pareto scaled before performing principal component analysis (PCA) and orthogonal partial least squares-discriminant analysis (OPLS-DA). The robustness of the created models was evaluated by monitoring the fitness of model (R^2^) and predictive ability (Q^2^) values. Models that yielded large R^2^ (close to 1) and Q^2^ (> 0.5) values were considered acceptable models [31]. Significant mass ions that were responsible for the class separation between the compared groups in the OPLS-DA scores plot were selected based on variable importance for projection (VIP) score greater than 1.0 [32]. Mass ions with FDR-corrected *p*-value less than 0.05 and VIP greater than 1.0 were selected as potential biomarkers and were considered significant. 

The significantly altered identified metabolites based on the uni- and multivariate analysis were imported to MetaboAnalyst version 4.0 to visualize the affected metabolic pathways. MetaboAnalyst software was used to perform binary comparison of the levels of the identified metabolites in the treated groups compared to controls using volcano plots and applying FDR-corrected *p*-values of less than 0.05 and fold changes greater than 2 (or less than 0.5). Additionally, hierarchical clustering analysis [33] was conducted and the level of biologically relevant significantly altered metabolites was visualized using heatmap graphical representations. 

## 3. Results 

### 3.1. GNRs Synthesis and Characterization 

GNRs of different surface chemistries, PEG-GNRs, and phospholipid-GNRs of aspect ratio ~4 were synthesized and fully characterized as described previously [21]. Briefly, both modified GNRs demonstrated typical optical spectra of two distinguished peaks; the transverse and the longitudinal peaks with excellent colloidal stability. PEG and phospholipid moieties were conjugated into the surface of the GNRs via thiol terminal as gold has a well-known high affinity toward thiol. The average length and width of the modified GNRs were in the range of 61 to 78 nm and 14 to 16 nm, respectively. The effective surface charges of PEG-GNRs and phospholipid-GNRs were +0.82 mV and −14 mV, reflecting successful surface conjugation. The surface conjugation of phospholipid into GNRs was confirmed by ^1^H-NMR, FT-IR spectroscopy, and TGA as described previously [21,22].

### 3.2. Mass Ion Detection and Metabolites Identification

**Figure 1:**
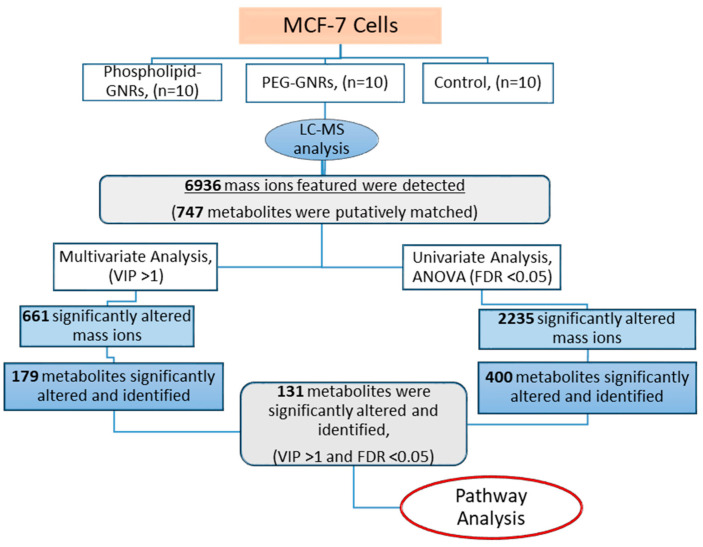
Workflow followed to investigate the effect of the two structurally modified gold nanorods (GNRs) (phosphorylated-GNRs and PEG-GNRs) on the cellular metabolites of MCF-7 cells using LC-MS-based metabolomics approach.

Figure 1 represents the workflow to investigate the influence of the two structurally modified GNRs on the metabolome of MCF-7 using LC-MS-based metabolite profiling. LC-MS analysis of cellular extracts from treated (phospholipid-GNRs and PEG-GNRs) and untreated (controls) MCF-7 cells detected 6936 mass features in both positive and negative ionization modes (Figure 1). The CV% of the QC pooled samples was less than 30% for more than 70% of the detected mass ions reflecting stable LC-MS analysis throughout the whole run. 

**Figure 2:**
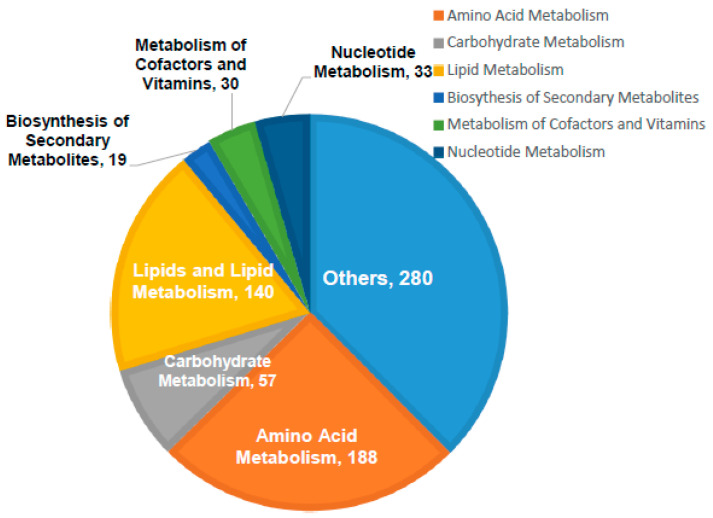
General classification of the identified metabolites according to their biological role. Large number of the identified metabolites are involved in amino acids, carbohydrate, lipids, and nucleotide metabolisms. The others identified metabolites are linked to diverse cellular processes including energy metabolism, redox homeostasis, and the biosynthesis of secondary metabolites.

Out of the 6936 detected mass ions, a total of 747 metabolites were putatively identified; 73 metabolites were identified based on accurate masses and retention times of the authentic standards analyzed under the same analytical conditions, while the remaining were putatively matched using IDEOM database based on mass accuracy and predicted retention times. The identified metabolites were involved in various metabolic pathways particularly amino acids metabolism, lipid biosynthesis and metabolism, and carbohydrate and nucleotide metabolisms as shown in Figure 2.

**Figure 3:**
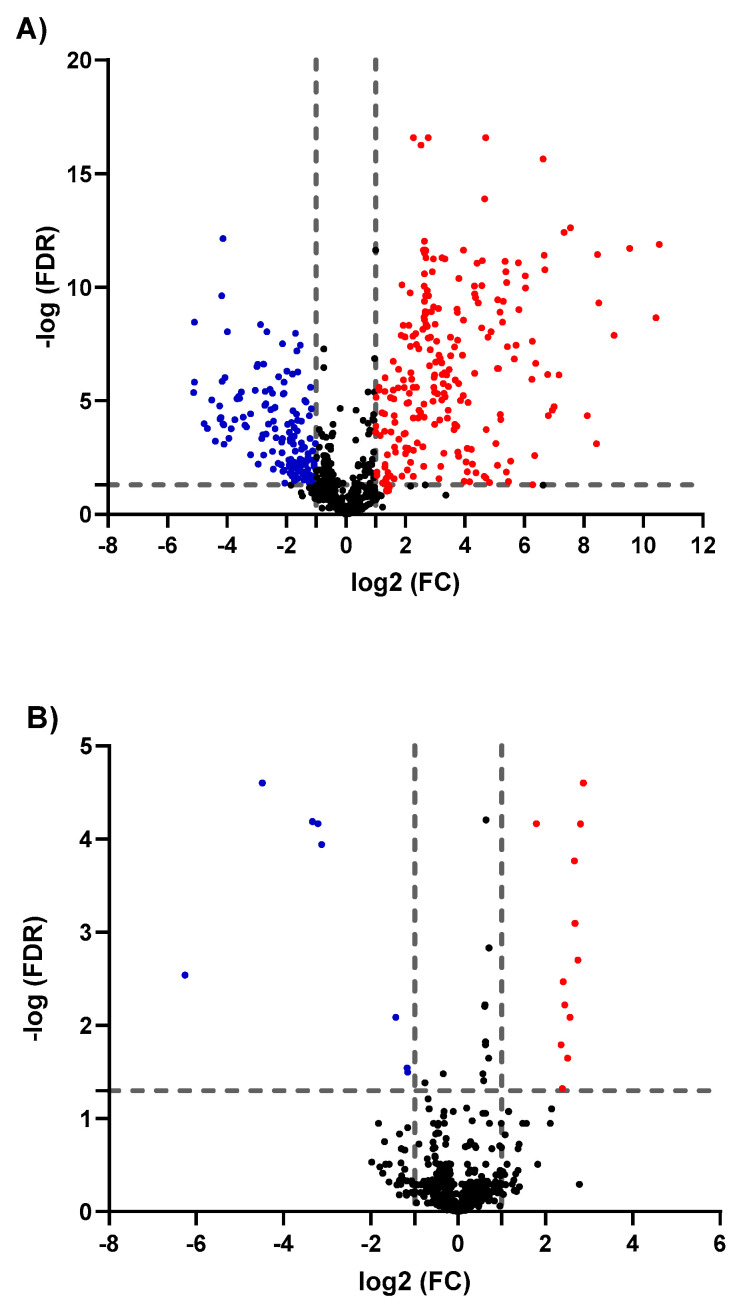
Volcano plots show the statistically significant altered metabolites (false discovery rate (FDR)-corrected *p*-value < 0.05, and fold change (FC) > 2 or < 0.5) in phospholipid-GNRs (**A**) and PEG-GNRs (**B**) treated MCF-7cells compared to controls. Significantly up- and downregulated metabolites are presented in red and blue circles, respectively.

To evaluate significantly up- or downregulated metabolites as of GNRs exposure, binary comparisons of the levels of the 747 identified metabolites were visualized in volcano plots applying FDR-corrected *p*-values (y-axis) and fold change (FC) (x-axis) thresholds of 0.05 and 2/0.5, respectively, as shown in Figure 3. The volcano plots depicted considerable metabolic differences. When compared to untreated MCF-7 cells, both phospholipid- and PEG-GNRs induced changes in the level of the identified metabolites with the former having significantly higher impact on cellular metabolism compared to the latter (Figure 3A,B, respectively). Phospholipid-GNRs resulted in more pronounced effect in the expression level of metabolites with 230 and 159 metabolites being significantly up- and downregulated, respectively, when compared to controls (Figure 3A). On the other hand, treating the cells with PEG-GNRs affected only 20 metabolites of which 12 metabolites showed an increase in their level compared to the untreated cells as shown in Figure 3B. 

### 3.3. Identification of Significantly Altered Metabolites Using Uni- and Multivariate Analyses 

In order to identify significantly altered metabolites, the metabolic expression of the three cellular extracts were compared using uni- and multivariate analyses (Figure 1). Results of significantly altered mass ions obtained from univariate (FDR-corrected *p*-value <0.05) and multivariate analyses (VIP>1) were combined to identify significantly changed compounds that can act as potential biomarkers.

**Figure 4:**
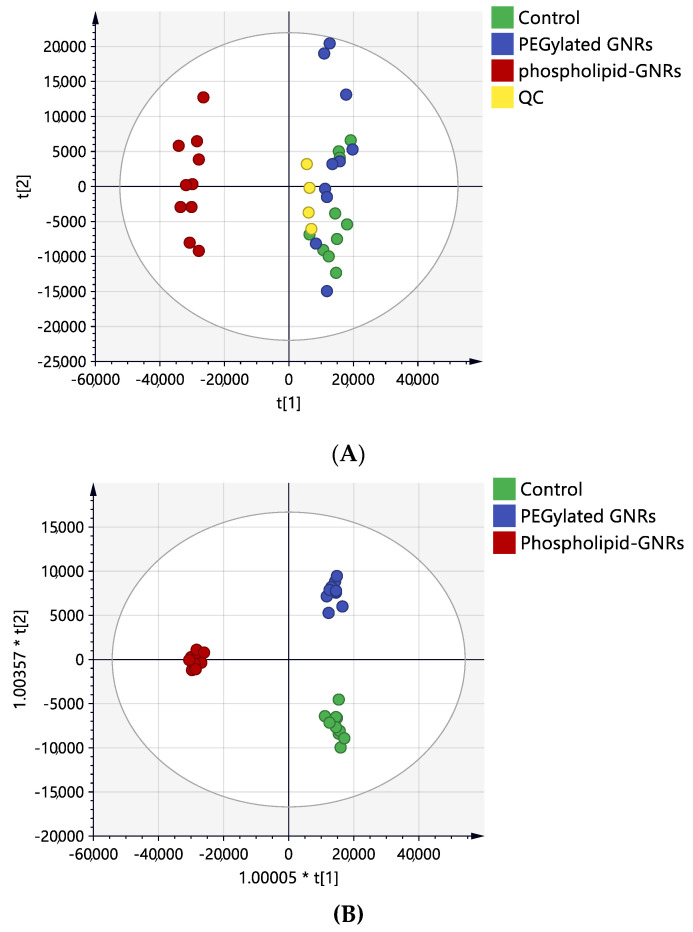
Scores plots of MCF-7 cells samples after 4-h treatment with PEG-GNRs (blue circles) and phospholipid-GNRs (red circles). Control group (green circle) without any treatment. (**A**) Principal component analysis (PCA) with quality control (QC) samples (R_2_X = 0.74, Q^2^ = 0.57, n = 10), (**B**) OPLS-DA (R2X = 0.72, R2Y = 0.98, Q2 = 0.89, CV-ANOVA *p*-value 2.2 × 10^−14^, n = 10).

Univariate ANOVA revealed significant perturbations in the levels of 2235 mass features between the three study groups of which 400 were putatively identified (Figure 1). For multivariate analysis, PCA and OPLS-DA models were generated to visualize any grouping or clustering of the data that can be consistently related to cellular metabolic changes (Figure 4A,B, respectively). Initially, the unsupervised PCA scores plot was obtained to give an unbiased overview of any possible trend or grouping in the sample’s datasets and to identify possible outliers (Figure 4A). The figure showed clustering of the pooled QC samples in the center of the scores plot, reflecting the stability of the LC-MS system throughout the run. MCF-7 cells treated with phospholipid-GNRs were clearly separated from the two other groups: PEG-GNR-treated cells and controls. The latter two groups did not show distinct separation and samples were overlapped (Figure 4A) highlighting the larger impact on cellular metabolites upon treating the cells with phospholipid-GNRs compared to PEG-GNRs.

The supervised OPLS-DA model displayed clear separation of the three sample groups and the scores plot yielded a satisfactory fitness of the model value (R2Y = 0.95) and a predictive ability value (Q2 = 0.87). As shown in Figure 4B, the ten biological replicates from each sample group were tightly clustered, reflecting reproducible data sets. A total of 661 mass ions had VIP values greater than 1, and therefore were responsible for the significant separation of the three groups in the OPLS-DA model. 

Results of significantly altered mass ions obtained from uni- and multivariate analyses were combined to yield a total of 493 mass ions with VIP >1 and FDR <0.05, of which 131 metabolites were putatively identified (Table S1, Supplementary). The significantly perturbed metabolites can act as potential biomarkers and, therefore, were exported for pathway analysis. 

### 3.4. Significantly Altered Metabolic Pathways 

**Figure 5:**
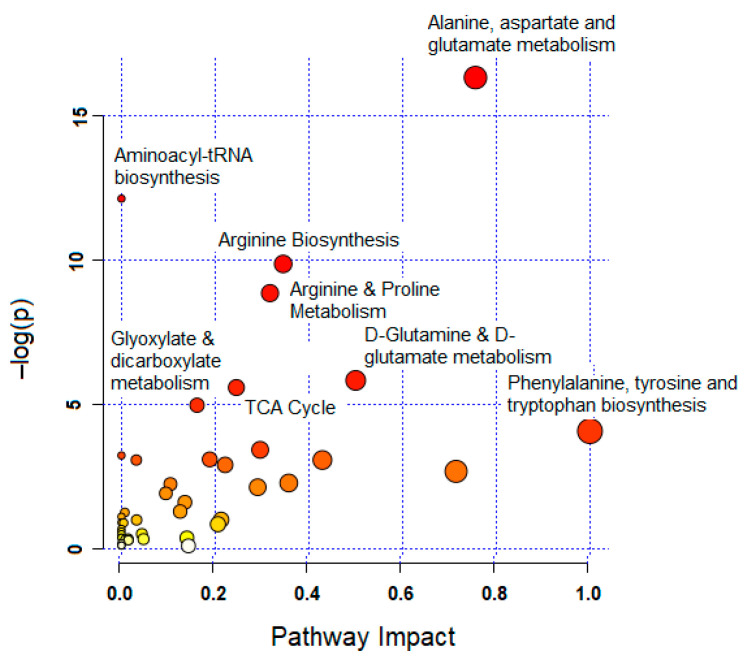
Summary of pathway analysis with MetaboAnalyst 4.0. The node color and size are based on its *p*-value and pathway impact value.

**Figure 6:**
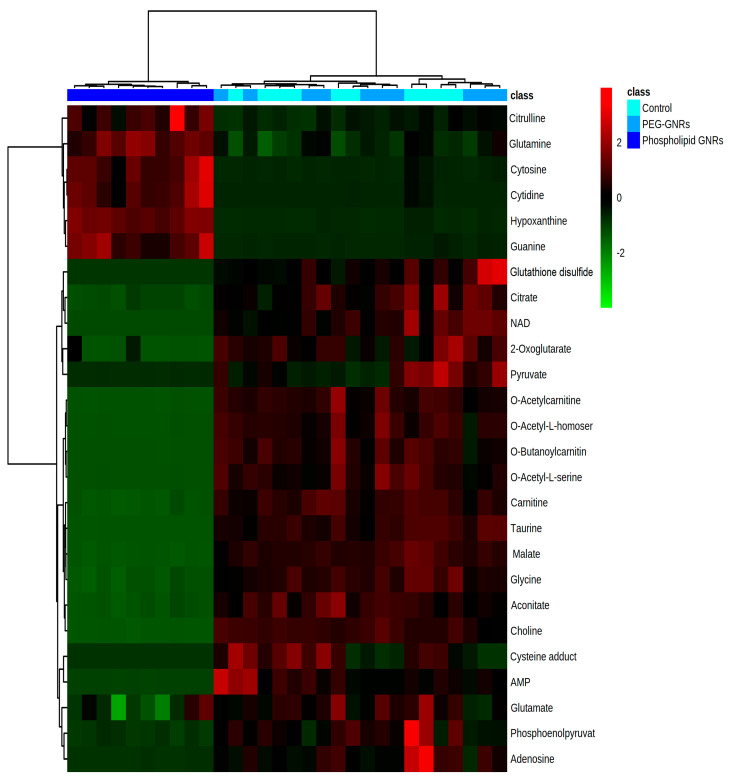
Hierarchal clustering and heatmap analysis of biologically relevant and significantly altered metabolites. Red and green colors refer to significantly up- and downregulated metabolites, respectively.

Treating MCF-7 cells with GNRs, particularly phospholipid-GNRs, resulted in significant perturbations in the levels of 131 metabolites associated with several metabolic pathways (Figure 5 and Table S1, Supplementary). Among the disturbed pathways are alanine, aspartate, and glutamate metabolism; aminoacyl-tRNA biosynthesis; arginine biosynthesis; and tricarboxylic acid cycle (TCA cycle, also referred to as the Krebs cycle or the citric acid cycle (CAC)) as can be seen in Figure 5. As we cannot rule out the effects of the structural modifications of GNRs on all 131 detected cellular metabolites, we focused on biologically relevant metabolites involved in important pathways linked to cancer cells growth and proliferation, and on those previously linked to toxicity mechanisms in cancer cells as reported in the literature. Therefore, Table 1 summarizes the significantly altered metabolites upon treating the MCF-7 cells with GNRs. The levels of these metabolites in the three study groups were visualized using heatmap (Figure 6) which showed a clustering of phospholipid-GNRs group with evident changes in the metabolite levels.

Metabolomics data revealed a substantial impact of phospholipids-GNRs on the metabolic profile of MCF-7 cells compared to PEG-GNRs which did not demonstrate significant effect as shown in Figure 6 and Table 1. The levels of metabolic intermediates and end-products associated with cellular energy metabolisms were significantly decreased by phospholipid-GNRs treatment. This includes four of the mitochondrial TCA intermediates (citrate, aconitate, 2-oxoglutarate (also known as α-ketoglutarate), and malate), metabolites involved in glycolysis (pyruvate and phosphenolpyruvate), and fatty acid β-oxidation (carnitine and acyl fatty acids). Nicotinamide adenine dinucleotide (NAD^+^) and oxidized glutathione (GSSG), which are both linked to cellular redox homeostasis, were not detected with phospholipid-GNR-treated group, indicating a significant reduction in their levels due to the exposure to phospholipid-GNRs. Additionally, the levels of two of the three building blocks of glutathione—cysteine and glycine—were also significantly decreased in phospholipid-GNRs group.

Pyrimidine and purine nucleotides metabolisms were among the most significantly altered biochemical processes in phospholipid-GNR-treated cells. The levels of cytosine and cytidine (involved in pyrimidine nucleotide metabolism) alongside hypoxanthine and guanine (involved in purine nucleotide metabolism) were found to be significantly increased, while adenosine monophosphate (AMP) and uridine monophosphate (UMP) were decreased in the same group. Furthermore, several essential and non-essential amino acids (glutamine, glutamate, arginine, alanine, and citrulline) that are vital elements for cell growth were perturbed in phospholipid-GNRs group.

**Figure 7:**
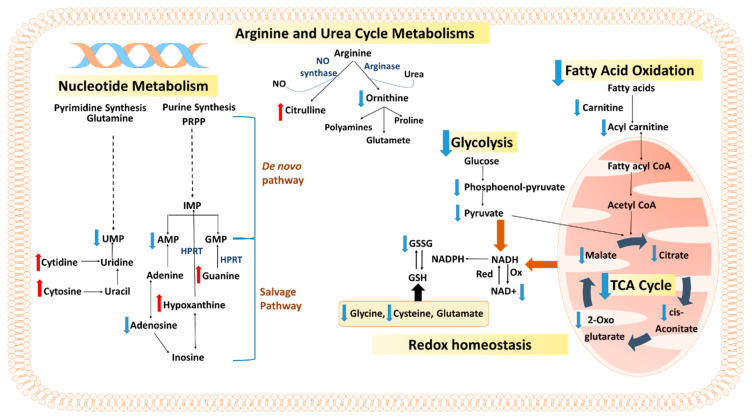
Overview of the affected pathways in MCF-7 cells after treatment with phospholipid-GNRs. Red and blue arrows represent increased and decreased levels, respectively. Adenosine monophosphate (AMP), guanosine monophosphate (GMP), inosine monophosphate (IMP), uridine monophosphate (UMP), 5-phospho-α-D-ribosyl 1-pyrophosphate (PRPP), hypoxanthine-guanine phosphoribosyltransferase (HPRT), nicotinamide adenine dinucleotide (NAD), glutathione (GSH), oxidized glutathione (GSSG), and nitric oxide (NO).

Schematic representation of the affected metabolic networks in MCF-7 cells after treatment with phospholipid-GNRs is shown in Figure 7.

## 4. Discussion 

GNP-induced cytotoxicity and antiproliferative effect in cancer cells are subjects of interest for clinical and GNP related researches. However, there are limited studies that investigated the mechanism of cytotoxicity and the metabolic consequences associated with the exposure to GNPs and how they influence the levels of cellular metabolites. Studies that explore the cellular molecular effects of GNPs on cancer cells are highly needed to elucidate the possible targets of these treatments for future designs of therapy. We previously investigated the cytotoxicity and the cellular death modality of phospholipid-GNRs and PEG-GNRs towards MCF-7 breast cancer cell line, and indicated that phospholipid-GNRs demonstrate drastic cytotoxicity and massive cellular uptake (as nanoaggregates) towards MCF-7 cells compared to PEG-GNRs [21]. In this study, we used LC-MS-based metabolomics approach, for the first time, as a comprehensive and robust analytical strategy to explore the metabolic changes in MCF-7 cells induced by the two structurally modified GNRs; PEG-GNRs and phospholipid-GNRs, at sub-toxic concentration. MS-based metabolomics study provides several advantages including high sensitivity and a wide range of metabolite coverage.

The fold changes in metabolite levels noticed herein (Table 1 and Figure 6) confirm that phospholipid-GNRs had larger impact on cellular metabolism, reflecting higher toxicity of this structural modification compered to PEG-GNRs, which is in line with our previous report [21]. The previously reported larger cellular uptake of phospholipid-GNRs by MCF-7 cells compared to PEG-GNRs has also contributed to the cytotoxicity of phospholipid-GNR [21].

Metabolic activities rely on the consumption of energy in the form of adenosine triphosphate (ATP), which is primarily generated, in normal cells, by mitochondrial oxidative phosphorylation (TCA cycle) and to a lower extent by glycolysis. Typically, cancer cells mainly use glycolysis for energy production rather than oxidative phosphorylation, a phenomenon referred to as “Warburg effect” [34]. The metabolomics data in this study suggest that phospholipid-GNRs altered the cellular energy metabolism of MCF-7 cells by distressing the TCA cycle and glycolysis (Figure 7). The TCA cycle operates in the mitochondrial matrix and has a starring role in the process of energy production. It is responsible for the generation of the reducing equivalents (NADH and flavin adenine dinucleotide (FADH2) reduced forms) that transfer electrons to the electron transfer chain to ultimately generate ATP in a process referred to as oxidative phosphorylation [35]. Although glycolysis plays a crucial role in cancer energy metabolism, oxidative phosphorylation can still largely contribute to energy production in some cancers such as breast cancer [36]. Oxidative phosphorylation has been reported to contribute for 91% of the total ATP production in breast carcinoma MCF-7 cells [37]. The decrease in the levels of citrate, aconitate, α-ketoglutarate, and malate in the current work clearly states that phospholipid-GNRs caused mitochondrial impairment resulting in TCA cycle dysfunction (Figure 7). The decrease in the levels of those four metabolites might indicate a reduction in the activity of citrate synthase, α-ketoglutarate dehydrogenase (α-KGDH), and fumarate hydratase (FH), which in turn might lead to diminished formations of cellular NADH and mitochondrial ATP and decreased oxygen consumption by the respiratory chain. The finding that phospholipid-GNRs caused mitochondrial impairment is in agreement with our previous results where the phospholipid-GNRs significantly disrupted the mitochondrial membrane potential and resulted in a considerable internalization of monomeric JC-1 in the cytosol [21].

Besides the TCA, metabolomics data suggest that phospholipid-GNRs inhibited glycolysis as pyruvate (end product of glycolysis that is introduced into the TCA cycle to fuel oxidative phosphorylation [34]) and phosphoenolpyruvate were significantly decreased (Table 1, Figure 7). Such effect might be a consequence of decreased ATP production due to TCA dysfunction as the early steps of glycolysis are ATP-dependent. Both PEG- and phospholipid-GNRs have negatively affected the levels of phosphoenolpyruvate and pyruvate in treated MCF-7 cells; however, this effect was more pronounced and significant with phospholipid-GNR-treated cells compared to controls (Table 1). While glycolysis yields less ATP than oxidative phosphorylation, it generates ATP in a faster rate suited with the high energy demands needed for the rapid and the abnormal proliferation and growth of cancer cells [38].

It is well known that cancer cells have the ability to rewire their metabolism to sustain the production of energy needed for rapid cell proliferation [39]. Fatty acids can provide an important energy source through β-oxidation and the production of acetyl-CoA [40]. Interestingly, phospholipid-GNR showed a downregulated fatty acid oxidation activity which was manifested as a decrease in the levels of carnitine and several acyl fatty acids (Table 1 and Figure 7). The negative perturbations of the TCA cycle and glycolysis activities, and the inability of MCF-7 cells to switch towards fatty acid oxidation, culminated in an energy crisis in phospholipid-GNR-treated cells and, consequently, unfavorable cancer cell growth. The previous findings have directly contributed to the high cytotoxicity that we reported previously with phospholipid-GNR against MCF-7 [21].

Cytotoxicity of NPs is dependent on several factors such as size and surface chemistry; however, the results are highly conflicting. A recent study by Gallud et al. [41] demonstrated that cationic modified GNPs caused more cellular toxicity compared to neutral or negatively charged NPs, where they localized in mitochondria of THP-1 cells and resulted in mitochondrial dysfunction. Interestingly, Kwon et al. [42] demonstrated that DSPE-PEG-triphenylphosphonium-coated ceria NPs showed high localization into mitochondria with great reactive oxygen species scavenging ability. On the other hand, the metabolic changes in HepG2 liver cancer cell line were studied upon treatment with gold nanospheres conjugated with three different chemical moieties: citrate, polyvinylpyrrolidone (PVP), and poly(styrenesulfonate) (PSS). The results revealed that metabolites related to ATP production were decreased significantly in the cells that were treated with negatively charged citrate-GNP and PSS-gold nanospheres [43]. In our current study, the surface chemistry of GNRs plays a crucial role in their cytotoxicity against breast cancer cells. As described in our previous study [21], we propose that decorating the NPs with phospholipid moiety enhanced their attachment to the cell membrane of cancer cells (which are rich in phosphatidylethanolamines (PE) phospholipids), and consequently their cellular uptake resulting in impactful effects on cellular apoptotic and metabolic pathways.

Another key energy metabolite that was reduced in phospholipid-GNR-treated cell is NAD+. High NAD+ level boosts glycolysis and fuels cancer cells [44], though NAD+ metabolism is implicated in cancer pathogenesis beyond energy metabolism. NAD+ regulates DNA repair mechanisms and gene expression and stability [45]. Therefore, decreased levels of NAD+ can considerably alter central biochemical processes required to promote the survival of cancer cells particularly against anticancer agents [44]. 

Cellular redox homeostasis is connected to energy metabolism. The ratio of NAD+/NADH plays a significant role in redox reactions for a number of energy-related and regulatory processes [46]. NADPH, produced in the mitochondria, is essential to restore oxidized glutathione (GSSG) to its reduced state (GSH). Glutathione (GSH), a tripeptide composed of glutamate, cysteine, and glycine, is fundamental in regulating cellular redox balance [47,48], and the ratio of GSH/GSSG is a reliable indicator of antioxidant capacity and vulnerability to oxidative damage [49]. Treating MCF-7 cells with phospholipid-GNRs created imbalance redox status and altered cysteine metabolism. GSSG was markedly reduced with phospholipid-GNRs mainly due to mitochondrial impairment, and thus GSH could not be recycled from GSSG nor synthesized from its building blocks; levels of cysteine and glycine were significantly deceased (Table 1, Figure 7). 

Metabolomic studies directed into the identification of biomarkers for apoptosis in cancer cell lines have reported a decrease in the levels of choline and taurine [50,51]. In the present study, the levels of both metabolites were significantly decreased in phospholipid-GNRs group suggesting that this GNR may promote cancer cell death through apoptosis. This finding is in line with our previous study where apoptosis was the main cellular death modality of MCF-7 cells exposed to phospholipid-GNRs via regulating the expression of several genes involved in the apoptotic events [21]. Therefore, our results provide an additional evidence for the use of choline and taurine as biomarkers for apoptosis in cancer cells as suggested by previous metabolomics studies [50,51].

Perturbations in nucleotide metabolism was noticed with phospholipid-GNRs cells. Purines and pyrimidines are essential components for DNA replication and the production of RNA and, consequently, cell proliferation. Both nucleotides are synthesized by the complementary salvage and de novo biosynthetic pathways (Figure 7). The salvage pathway in purine metabolism recycles the degraded bases such as hypoxanthine and guanine, with the help of hypoxanthine-guanine phosphoribosyltransferase (HPRT), to generate inosine monophosphate (IMP) and guanine monophosphate (GMP), respectively [52]. In cancer cells, the de novo biosynthetic pathway is reinforced to generate IMP in highly conserved enzymatic steps which ultimately will contribute to the production of AMP, GMP, adenosine, and inosine [52,53], as shown in Figure 7. The findings herein showed that phospholipid-GNRs could interfere with DNA replication and synthesis in MCF-7 cells by enhancing purine catabolism and suppressing both the salvage pathway (high levels of hypoxanthine and guanine were detected) and the de novo biosynthetic pathway (decreased levels of adenosine and AMP were noticed). Similarly, increased cytosine and decreased UMP levels in phospholipid-GNRs treated cells pointed to suppression to the salvage and de novo biosynthetic pathways in pyrimidine metabolism, respectively [53]. Interestingly, PEG-GNRs group showed decreased levels of guanine, cytidine, and cytosine, reflecting mainly activation of the salvage pathway in purine and pyrimidine metabolism.

Literature has supported the importance of amino acids in cancer metabolism and has linked them to diverse array of processes vital for cell proliferation including protein and nucleotides biosynthesis, maintenance of cellular redox homoeostasis, and synthesis of the TCA intermediates [54]. Phospholipid-GNRs induced pronounced effects on the levels of several amino acids involved mainly in arginine and urea cycle metabolisms (noted by the alterations in the levels of arginine, citrulline, and ornithine), and glutamine metabolism (reflected by the high and the low levels of glutamine and glutamate, respectively) as can be seen in Table S1 and Figure 7. The effect of phospholipid-GNRs on arginine and urea cycle metabolisms might point to altered levels of arginine metabolism enzymes, mainly nitric oxide synthase and arginase (Figure 7). The former enzyme is responsible for the production of citrulline and nitric oxide while the latter produces urea and ornithine which is further used for the production of polyamines essential for cell proliferation and metastasis [55]. Alongside its role in urea synthesis and maintaining nitrogen balance, arginine has been identified as necessary for both cancer cell growth and normal immune function [55]. Recent studies in mouse breast cancer models revealed inhibition of tumor growth and enhancement of immune responses with arginine supplementation [56]. Notably, the major metabolic route affected by arginine and urea cycle metabolisms in cancer is nucleic acid synthesis, mainly pyrimidine synthesis [57], which is also confirmed by the effect of phospholipid-GNRs on MCF-7 cells in the current study. 

With regards to glutamine metabolisms, it is well known that cancer cell metabolism depends on glutamine for proliferation and growth. Glutamine and glutamate play major roles in cellular energetics and redox homeostasis, de novo purine and pyrimidine synthesis, and serve as precursors for glutathione and α-ketoglutarate [53,58]. It is expected that phospholipid-GNR might result in dysregulation of enzymes necessary to convert glutamine to glutamate, including the two mitochondrial glutaminases (GLS and GLS2) and consequently could affect diverse functions in cancer cell metabolism. A study conducted by Huang et al. [59] revealed that exposing human dermal fibroblasts cells to GNPs resulted in significant alterations in glutamate and glutathione metabolism pathways.

Note that alterations in cellular metabolic pathways have a large impact on the distinctive growth pattern of cancer cells. The malfunctions phospholipid-GNRs triggered in several crucial cellular biochemical processes essential for the survival and the proliferation of the cancer cells might highlight the importance of considering metabolic modulation as a promising therapeutic target in cancer therapeutics.

## 5. Conclusions 

Global metabolites profiling using an LC-MS-based metabolomics approach was used to investigate the metabolic abnormalities in breast MCF-7 cell lines when exposed to sub-toxic concentrations of two structurally modified GNRs: phospholipid-GNRs and PEG-GNRs. Metabolomics data revealed that phospholipid-GNRs resulted in more significant and pronounced impact on the cellular biochemical pathways than PEG-GNRs due to their surface chemistry. Phospholipid-GNRs induced perturbations in several crucial metabolism pathways essential for the normal growth and proliferation of cancer cells. This included a dysfunction in TCA cycle, a downregulation in glycolytic activity, and imbalance in the redox state which cumulatively led to inadequate ATP production. Additionally, impairment in nucleotide metabolism and altered levels of amino acids pool were noticed when cells were treated with phospholipid-GNRs. The effects of PEG-GNRs were limited to alteration of glycolysis and pyrimidine metabolism. The current study shed light on the importance of metabolomics as a valuable analytical technique to explore the mechanism of action of GNRs of different surface chemistry, provide holistic insights into their effect on cellular metabolic pathways, and highlight metabolic targets that might serve as promising treatment strategy in cancer. Analysis of non-aqueous metabolites will be conducted in a future lipidomics study to thoroughly investigate the effect of the two GNRs on the lipid profile of breast MCF-7 cell line.

## Figures and Tables

**Table 1:** Biologically relevant metabolites significantly changed in MCF-7 cells after treatment with phospholipid- and PEG-GNRs.

Mass (Da)	RT (min)	Formula	Putative Metabolite	IDC *	Control	Phospholipid-GNRs **	PEG- GNRs **
**TCA cycle**							
174.0164	11.30	C_6_H_6_O_6_	Aconitate	L2	1.00	0.18	1.01
146.0215	10.37	C_5_H_6_O_5_	2-Oxoglutarate	L1	1.00	0.13	0.95
192.0270	11.62	C_6_H_8_O_7_	Citrate	L2	1.00	0.21	1.18
134.0215	10.51	C_4_H_6_O_5_	Malate	L1	1.00	0.10	0.96
**Glycolysis**							
167.9824	11.30	C_3_H_5_O_6_P	Phosphoenol-pyruvate	L2	1.00	0.08	0.47
88.0161	10.35	C_3_H_4_O_3_	Pyruvate	L2	1.00	0.11	0.77
**Fatty Acid Oxidation**							
161.1052	9.71	C_7_H_15_NO_3_	Carnitine	L1	1.00	0.15	0.94
203.1158	8.55	C_9_H_17_NO_4_	O-Acetylcarnitine	L1	1.00	0.03	0.90
231.1471	7.51	C_11_H_21_NO_4_	O-Butanoyl-carnitine	L2	1.00	0.01	0.77
**Redox Homeostasis and Apoptosis**							
663.1097	9.53	C_21_H_27_N_7_O_14_P_2_	NAD+	L2	1.00	0.00	0.99
306.0760	10.85	C_20_H_32_N_6_O_12_S_2_	Glutathione disulfide	L1	1.00	0.00	1.61
191.0252	10.17	C_6_H_9_NO_4_S	a Cysteine adduct	L2	1.00	0.00	0.55
75.0320	11.21	C_2_H_5_NO_2_	Glycine	L1	1.00	0.22	0.85
147.0531	7.55	C_5_H_9_NO_4_	O-Acetyl-L-serine	L2	1.00	0.02	0.91
161.0688	7.09	C_6_H_11_NO_4_	O-Acetyl-L-homoserine	L2	1.00	0.03	0.93
103.0997	10.16	C_5_H_13_NO	Choline	L2	1.00	0.04	0.98
125.0147	10.82	C_2_H_7_NO_3_S	Taurine	L1	1.00	0.01	0.90
**Amino Acid Metabolism**							
175.0957	11.12	C_6_H_13_N_3_O_3_	Citrulline	L1	1.00	2.17	1.07
146.0690	10.67	C_5_H_10_N_2_O_3_	Glutamine	L1	1.00	1.67	1.13
147.0531	9.97	C_5_H_9_NO_4_	Glutamate	L1	1.00	0.74	0.90
**Nucleotide Metabolism**							
267.0968	7.95	C_10_H_13_N_5_O_4_	Adenosine	L1	1.00	0.01	0.58
347.0629	9.30	C_10_H_14_N_5_O_7_P	AMP	L1	1.00	0.01	1.05
111.0433	9.42	C_4_H_5_N_3_O	Cytosine	L1	1.00	12.23	0.65
243.0856	9.47	C_9_H_13_N_3_O_5_	Cytidine	L1	1.00	25.42	0.00
136.0384	8.78	C_5_H_4_N_4_O	Hypoxanthine	L1	1.00	17.55	0.79
151.0494	9.99	C_5_H_5_N_5_O	Guanine	L2	1.00	35.42	0.50

* IDC: ID confidence; metabolite identification level according to metabolomics standards initiative L1—Level 1: metabolites with accurate masses and retention times that matched the corresponding analyzed authentic standards, L2—Level 2: metabolites were putatively identified based on the accurate mass and the predicted retention times. ** Phospholipid and PEG-GNRs: values represent fold change of each metabolite in phospholipid- and PEG-GNR-treated cells against control.

## Data Availability

The data presented in this study are available in the supplementary material.

## Supplementary Materials

The following are available online at https://www.mdpi.com/2218-273X/11/3/364/s1. Table S1: List of metabolites significantly altered in MCF-7 cells after treatment with gold nanorods (GNR). IDC: metabolite identification level according to the metabolomics standards initiative L1; Level 1, L2; Level 2. Pos and Neg indicate positive and negative modes of ionization, respectively. Colors in the table represent changes in metabolite levels with blue indicating a decrease and red denoting an increase.
